# Improved Wellbeing for Both Caretakers and Users from A Zoo-Related Nature Based Intervention—A Study at Nordens Ark Zoo, Sweden

**DOI:** 10.3390/ijerph16244929

**Published:** 2019-12-05

**Authors:** Eva Sahlin, Björn Johansson, Per-Olof Karlsson, Jenny Loberg, Mats Niklasson, Patrik Grahn

**Affiliations:** 1 Department of Work Science, Business Economics and Environmental Psychology, Swedish University of Agricultural Sciences, P.O.Box.88, SE-230 53 Alnarp, Sweden; patrik.grahn@slu.se; 2 Nordens Ark Foundation, Åby Säteri, SE-456 93 Hunnebostrand, Sweden; bjorn.johansson@nordensark.se (B.J.); pelle.karlsson@nordensark.se (P.-O.K.); jenny.loberg@nordensark.se (J.L.); mats.niklasson@nordensark.se (M.N.); 3 Department of Animal Environment and Health, Swedish University of Agricultural Sciences, P.O. Box 234, SE-532 23 Skara, Sweden; 4 Southern Swedish Forest Research Center, Swedish University of Agricultural Sciences, P.O. Box 49, SE-232 52 Alnarp, Sweden; 5 Gothenburg Global Biodiversity Centre, University of Gothenburg, Box 100, S-405 30 Gothenburg, Sweden

**Keywords:** sustainability, nature exposure, human–animal interaction, neurological disabilities, outdoor education, animal and nature course

## Abstract

Nature-based interventions have been proposed to promote physical and mental health and give stress reduction. Little attention has been given to the potential of zoos for human health and wellbeing. A disadvantaged group in Sweden regarding access to nature are individuals with disabilities who consequently do not have the same access to these health benefits as other groups. To increase awareness and knowledge regarding spending time in nature and with animals, courses directed at caretakers for persons with disabilities and their users were held at Nordens Ark, a zoo in Sweden. To explore if the courses had led to increased nature activities, and if participating in the courses had affected caretakers’ and their users’ health and wellbeing, questionnaires and interviews for evaluating the courses were used. The results showed improved quality in nature visits because of course participation as well as positive effects for the wellbeing, sustainability for the caregivers and users in their working lives, and relationships were positively affected. The conclusion from this study is that nature and animal-based education should be more frequent to provide opportunities for a disadvantaged group to have the positive effects of nature of which most other groups have obvious access to.

## 1. Introduction

In today’s society, lifestyle-related illnesses, such as stress-related mental illness, cardiovascular disease, and type 2 diabetes, constitute an increasing proportion of the population’s morbidity. Staying in natural environments has been proposed for over 30 years to promote health, and this has in particular been about preventing or curing lifestyle-related illnesses, e.g., by reducing high stress levels and increasing people’s ability to focus attention [1]. The research area is developing rapidly regarding issues aiming to document and explain the health effects of stays in natural environments. The positive effects of visits to natural environments on human health has now a solid scientific basis, where proximity and exposure of green areas during everyday life lead to better health and a longer life expectancy [2,3,4,5,6]. The improved health is explained by many interacting health-promoting factors, such as exposure to sunlight and fresh air, increased physical activity, and that the natural areas reduce high stress levels and increase executive function [1]. Another health promoting factor is of social character and embrace the effect of being together with people and sharing experiences e.g., positive activities in nature which was reported in Sahlin et al. [7].

Interacting with animals, for example in animal-assisted therapy, has been shown to have the potential to improve the health of the participants regarding several diseases and disorders, such as psychological disorders, anxiety, ASD (autism spectrum disorder), ADHD (attention deficit hyperactivity disorder), PTSD (Post-traumatic stress disorder), and dementia. This applies to alleviation of symptoms, improvement in social interaction and mood, reduced aggression, lower stress levels, blood pressure, and heart rate etc. With regard to bodily functions, improvements have been found regarding balance, pain relief, etc., in participants with, for example, MS (Multiple sclerosis), CP (Cerebral palsy), and traffic injuries [8,9,10,11,12,13,14,15,16].

Nature-based interventions (NBI) consists of many different therapeutic or health-promoting interventions, which may include gardening, wilderness hiking, or animal activities. The latter can be about riding horses, caring for cows or pigs, petting dogs, or just being in a context, e.g., a farm or a zoo, where they keep animals. For a large part of the modern urbanized populations, zoos may offer one of few possibilities for nature visits, and especially encounter of animals. Globally, zoos attract more than 700 million visits annually [17]. However, very little attention has been given to the potential of zoos for human health. Given the great number of visits to zoos, it is remarkable how relatively little zoos have been utilized in the field of human health. Swiss biologist Heini Hediger was a pioneer in the so-called zoo biology and claimed as early as the 1960s that humans and animals could have fruitful social contacts at the zoo [18,19]. It has also been found that zoo professionals and zoo animals establish human–animal relationships (HAR) and even human–animal bonds (HAB) [20]. The same is true when using the zoo in teaching, where you let disabled and sick students participate in the care of the animals [21,22]. In such cases, HAR and/or HAB appear to arise, and the intervention produces a successful result. However, it turns out that visitors to the zoo are also positively affected. Studies are rare but, in a few studies, positive effects of zoo visits on human health have been documented [23,24,25].

Several recent reviews show that NBI are effective in treating, rehabilitating, and caring for sick and disabled people, such as participants with schizophrenia, bipolar disorder, PTSD, ADHD, and ASD. Studies show for example that NBI leads to better psychological wellbeing, reduces high levels of stress, and improves coping ability and sleep quality in intervention groups but not in the control groups [26,27,28,29,30,31]. When it comes to animal-assisted interventions (AAI) specifically, systematic reviews and meta-analyses show that they provide significant effects for many different diseases and disabilities, not least regarding neurorehabilitation [32,33].

NBI is based on methods developed in several disciplines, such as occupational therapy, physiotherapy, nursing science, environmental psychology, veterinary medicine, animal welfare, landscape architecture, and horticulture [34]. Within NBI, various specialized professions have been developed, such as horticultural therapists, where they primarily work with garden activities, and equine therapists, where they work with horses [34]. However, many professions use NBI, especially in healthcare and education. A theory that has developed is that the treatment is based on support from both the physical environment (such as nature including wildlife), the social environment (other participants but also companion animals), and the cultural environment (activities, languages, knowledge transfer). In this context, the therapists must learn how to use the various forms of support provided by the environment [1,22,34,35,36,37].

A group that has a great responsibility to care for people with disabilities is personal caretakers. Their users often suffer from poor wellbeing. NBI could provide them with good tools in their everyday lives. To the best of our knowledge, there are no studies focusing on this group of professionals, whose everyday life is filled with demands. The zoo environment can hypothetically offer an interesting and attractive opportunity to give both caretakers and their users knowledge of what animals and nature can offer to improve wellbeing by visits in nature and outdoor activities. Could courses in such a setting, specifically aimed at caretakers, change the behaviour and motivation of the target group and their users? We wanted to examine if the courses had effects on their everyday lives (personal caretakers and their users). This includes if they participated in activities in nature. In addition, we wanted to see if it affected users’ wellbeing. However, few studies focus on the zoo as a health-promoting site. In a review of human–animal interactions, only 1 out of 24 papers on animals’ benefits to humans has been done in zoos and none of the 51 papers on the subject of animal-assisted intervention use a zoo setting [38].

### 1.1. Aim

The aim of this study was to elucidate the effect of zoo-related activities in combination with outdoor activities in a natural environment on the behavior, attitudes, and wellbeing of personal caretakers for persons with disabilities. The final aim was to lower the threshold and to encourage these personal caretakers to increase the frequency of outdoor activities and contact with animals for their users. This was done by offering the personal caretakers courses held at Nordens Ark Zoo, a Swedish zoo with a high profile in conservation and a well-developed infrastructure for outdoor activities.

### 1.2. Research Questions

- Did the nature- and animal-oriented course convey knowledge, inspiration, feelings of safety, and security in nature to participating personal caretakers and educators, leading to increased nature activities for their users?
- Did participation in the course affect health and wellbeing:
  - For the personal caretakers?
  - For their users?

## 2. Materials and Methods 

### 2.1. The Setting

**Figure 1:**
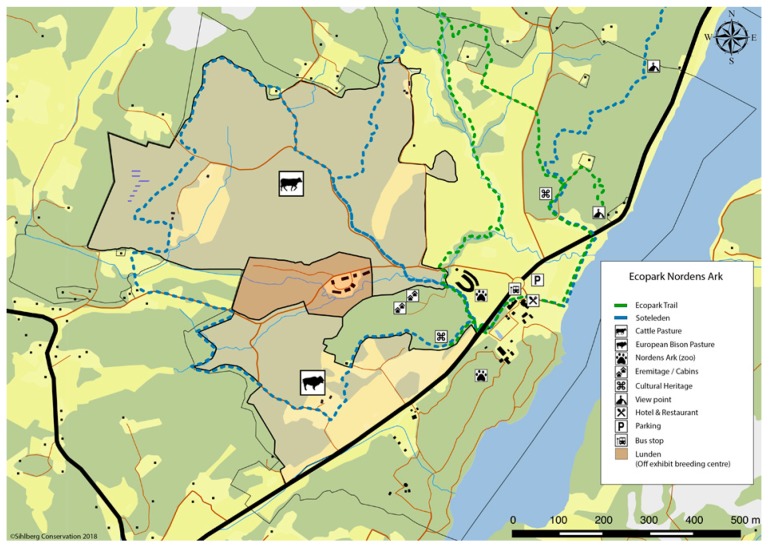
The Nordens Ark Zoo.

The private foundation Nordens Ark (https://en.nordensark.se/) was started in 1989 and is primarily focused on conservation of endangered species, education, and outreach. It is situated at the Swedish west coast, about 120 km north of Gothenburg and 200 km south of Oslo, the capital of Norway. A main goal of the foundation is to strengthen populations of endangered species by breeding, rearing, and when possible, reintroducing them to their natural environments. Nordens Ark Zoo is running a year-round open zoo where the conservation projects and endangered animals are displayed for the public. Annually approximately 120,000 visitors come to the zoo. Compared to conventional zoos, Nordens Ark Zoo stands out in several respects. The non-profit profile is visible in all activities and is notable also for the typical visitor coming only for the zoo. A large part of the budget/income comes from donations and testaments, which lays the foundation for an unusually strong commitment to breeding and conservation projects. Outreach and information to visitors emphasizes the high conservation profile of the park. The animals live in large enclosures where they should feel safe and comfortable, as it is important for the animals to thrive and reproduce. As a result, the animals can sometimes be difficult to observe. The different focus of Nordens Ark Zoo is well perceived among our visitors (Figure 1).

Nordens Ark Zoo is physically located in a region attractive for tourists, with a large number of summer visitors. The foundation owns about 400 hectares of land of which ca 70 ha is the publicly open zoo and the breeding centre. The remaining 330 hectares is agricultural land and forests, which since 2011 has been run as an Ecopark where ecological restoration, education, and research are the focus. The site is located in the nemoral natural geographic region, representing the main part of Western Europe, with forests of deciduous trees like European ash (*Fraxinus excelsior*), Norway maple (*Acer platanoides*), European oak (*Quercus robur*), and a limited presence of coniferous forests, mainly Norway spruce (*Picea abies*) and Scots pine (*Pinus sylvestris*) [39,40]. The zoo section is wooded and lies in a more hilly and rocky area of the property, while the remaining part is more and more transformed into pastureland. Many threatened native species of insects and birds that are dependent on a grazed landscape are disappearing from large areas of Sweden. To work against that trend and increase biodiversity, a large part of the former plantation forest is today converted into semi-natural grasslands using mainly cattle (*Bos tarus*), sheep (*Ovis aries*), and European bison (*Bison bonasus*) for grazing. Several facilities have been constructed in the Ecopark to promote visits and experiences of nature such as foot paths, information signs, camp sites, etc. Outreach and education activities is growing year by year in the Ecopark. Since the opening in 1989, the zoo and its animals in combination with the Ecopark surrounding the zoo have been the template for various educational and nature activities, primarily for primary schools as well as for groups of children with special needs. An important part of this activity has been interaction with animals, mainly with farm animals. 

### 2.2. The Courses

**Figure 2:**
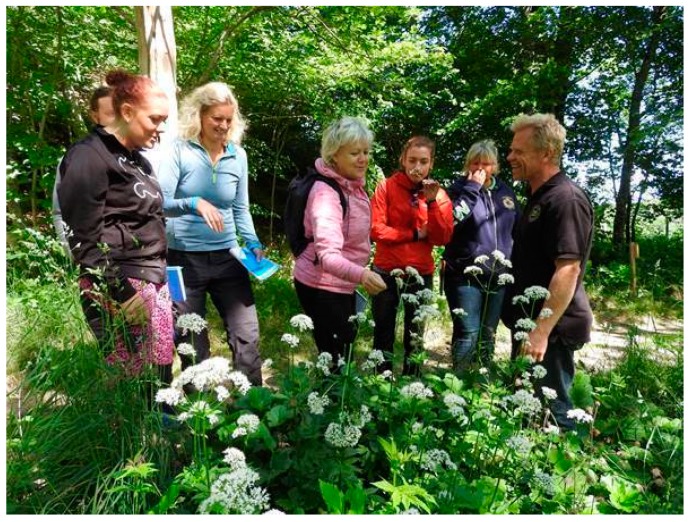
Botanical excursion with caretakers during one course.

During three years (2015–2018), courses for caretakers of persons with disabilities and their users were held at Nordens Ark Zoo. The focus was to increase awareness and knowledge regarding spending time in nature and being with animals. In the first step, 3-day courses (C1) were organized for caretakers including knowledge about nature and outdoor skills like making fire, cooking, sleeping outdoors, and interactions with animals. In a second step (C2), these caretakers came back together with their user/users to Nordens Ark Zoo for a 1-day or a 2-day course where they practiced their new knowledge with their user/users under the course leader’s supervision (Figure 2).

### 2.3. Course Activities

At the start of each course, a scientific presentation was given about the health effects of nature visits and animal interaction. This served as a base for further activities and discussions.

#### 2.3.1. Zoo-Related Activities

In all courses, zoo-related activities were included such as how to approach animals, general knowledge about animals and their behavior, and meeting and interacting with animals, both domestic animals in our farm and wild animals in our zoo.

#### 2.3.2. Outdoor Activities

All participants were taught basic outdoor skills, such as how to start a fire with materials from nature. Outdoor cooking over open fire and on portable stove was practiced. In all courses, basic navigation skills using map and a compass were taught as well as basic plant knowledge. In the second night of the course, participants slept outdoors in sleeping bags in shelters.

### 2.4. The Study Population

Inclusion criteria: The study population represented participants from all courses during the projects last year, with 35 persons in total. Most of the participants were working as caretakers/teachers employed in daily programs or schools directed at individuals with special needs, but also owners of private green care companies in Sweden. The course participants’ professional background varied: Teachers on different levels, some with specialist knowledge, assistant nurses, equine therapist, occupational therapist, field biologist, nature conservation consultant, bio-medical analyst, nurse, and students. Some of the participants lacked professional education for the specific user-group. Hereafter, all C1 participants are referred to as caretakers. The different caretakers’ programs embraced users with difficulties and disabilities in different physical and mental areas, however the majority had neuropsychiatric disorders (such as autism, ADHD, Huntington’s disorder) and mental retardations (Down’s syndrome) but also one user with CHARGE syndrome (a genetic disorder). In the course, a teacher responsible for pre-schoolchildren without any disabilities was also included. Hereafter, all are referred to as “users.” There were also a variety in severity concerning disabilities including users on extremely low level of development and more well-functioning individuals. Both sexes were represented among the users, however, in one group, there were only men. Twelve to 14 months after the course ended, all 35 caretakers received a 12-month follow-up questionnaire by email. Those who answered all or almost all questions were included in the study. All 35 caretakers returned the follow-up questionnaire. However, five were excluded because they had not been working with the target group during the last 6 months or because the questionnaires were only partly answered, thus the study population comprised of totally 30 course participants (28 females, 2 males).

#### Ethical Consideration

No ethical approval was requested for this study, according to the ethical board in Gothenburg, Sweden (A. Fredriksson, personal communication 20181017). However, to follow good ethical research standards. each caretaker and course participant were asked for their written consent to participate in the study, which was given from all participants.

### 2.5. Measures

The study has applied a mixed model design to give the possibility of a broad and deep analysis [41]:

- A follow up questionnaire 12–14 months after participation in the course (C1) at Nordens Ark Zoo. After each question, space for comments was included.
- Semi-structured exploratory interviews.

When the courses were designed, it was decided to evaluate the effects of the project. The course leaders and the first author, who followed the course during the whole project, constructed questionnaires for evaluating and follow-up of the participants’ experiences and evaluations of the course. The answers in the 12-month follow up questionnaire (A) were used and statistically analyzed in this study.

#### 2.5.1. Quantitative Measures

The quantitative results are based on the 12-month follow-up survey conducted 12–14 months after completing the course. The questionnaire included 10 questions concerning:

- Whether the caretakers used the activities and the knowledge gained during the courses in their work with their users; e.g., Have activities learned during the courses been practiced with users after the course? Have you developed new animal/nature activities after the course? (response alternatives were yes/no).
- Comparison of the frequency and quality/content of nature activities before and after the courses; e.g., *“Are the activities learned during the courses compared to before the courses practiced:* Fewer/more seldom; about the same frequency; more often.
- If participation in the courses had led to any changes in prioritization of activities with the users; e.g., Do you make other activity priorities after the course e.g., do you more often choose activities including animals and/or nature? (response alternatives were yes/no).
- If and to what extent the course had contributed to more inspiration and joy in their work and also for their own part; e.g., *To what extent have the courses been of importance for inspiration and joy in your work?* (Response alternatives 1 (of no importance at all) to 6 (of great importance).

Additionally were also questions about whether the course had influenced the users’ curiosity, interest, openness to new activities, and mood.

#### 2.5.2. Qualitative Measures

Included in the 12-month questionnaire was information on an upcoming research study and invitation to participate in an interview with questions concerning the participants’ experiences of the course. Twenty-eight participants reported interest in participating and 15 of them were included. The selection for the interview was stratified, based on geographical distances and reasonableness of travelling as well as a variety in the participants’ area of work [42] (see Table 1).

Evaluations and experiences of the course were explored through exploratory interviews. These were carried out using five broad themes:

(1) Experiencing the environment at Nordens Ark Zoo; e.g., How did you experience the different environments at Nordens Ark Zoo? How did you experience being out in nature almost 100% of the course?
(2) Knowledge contributing to your feelings of safety and security when performing outdoor activities with your users; e.g., Did the course convey knowledge that contributed to feelings of safety and security when taking your users out in nature or to encounter with animals?
(3) Practical knowledge and experiences from the course useful in your outdoor work with users; e.g., Did the course offered practical activities and experiences that could be adapted in your work at home with your users?
(4) Health and wellbeing benefits acquired from participation in the course; e.g., Do you find that being in nature has affected your users’ health and wellbeing in any way?
(5) Frequency and content of nature visits after the course; e.g., “Describe if and how your users’ attitude towards nature visits have changed after the course.

The explorative interviews were designed as conversations where the introductory thematic questions were followed up by questions in the style of “explain more closely; can you develop what you mean” [42,43]. The questions were sent in advance. The interviews lasted for 35–70 min. Five were held in the participants workplaces with group interviews of 2–3 caretakers at the time and four individual interviews were carried out by telephone. All interviews were conducted 14–18 months after completed courses. The interviews were recorded using Olympus WS-852 digital recorder and transcribed verbatim within a couple of weeks thereafter. The interviews were analyzed using latent content analysis [44].

### 2.6. Statistical Analyses

Data were analyzed based on the 12-month follow up questionnaires and presented by descriptive statistics. All statistical analyses were performed using IBM SPSS (Statistical Package for the Social Sciences) Statistics version 22 (SPSS Inc., Chicago, IL, USA).

### 2.7. Qualitative Analysis

The interviews were analyzed in several steps, inspired by the inductive content analysis method [44,45,46]. Each interview was repeatedly read to attain a thorough understanding of the intended meaning. Comments, summaries, and annotations were written and condensed into categories. Then, the categories were grouped and given headings. Finally, the headings were compared, which led to named themes. All interviews were analyzed by the first author. To secure trustworthiness, three randomly selected interviews were read by all authors and analyzed separately by the first and last authors. No discrepancies were found when the analyses were compared. Interviewees were asked to read and comment on their quotes to ensure correctness of intent and interpretation, as well as to agree to the use of the quote. All confirmed and agreed with our analyses. The procedures in the process followed established principles to ensure highest possible quality [47] and included peer debriefing, participants’ confirmation, and procedures for documentation to enable checking and re-checking the data during the entire process.

## 3. Results and Discussion

Four superordinate themes and 9 subordinate themes emerged from the interviews (see Table 2).

### 3.1. The Impact of the Environment

#### 3.1.1. The Course Environments

The course was enriched by offering experiences and activities in several different types of natural environments surrounding Nordens Ark Zoo, such as forests and hills, meadows, cliffs, and coastal environments, all typical in this part of Sweden [39,48,49]. Positive feelings of tranquility due to the impact of the environments were outspoken and highlighted by all caretakers. *“Hard to put my finger on the importance of the environment, but it conveyed peacefulness- the course days were like being in an indestructible bubble that no one could disturb.”* Extensive environmental psychological studies of the impact of natural environments on human health and wellbeing have resulted in that natural environments can be described based on eight perceived sensory dimensions (PSDs) [50]. Of these eight, serenity is the most in demand, and the one that in extensive studies has been found to have the greatest impact on stress reduction and tranquility [34,50].

All informants experienced the course environment as peaceful and relaxing. The interviews gave several descriptions about the soothing environmental impact that gave recovery from everyday stress especially during the first-step three-day course (C1) “*I forgot about what day it was*” and “*I find the environment fantastic. I felt a sense of calm - which I believe has a lot to do with the environment and the whole atmosphere.”* The experiencing of tranquility and stress relief in nature has earlier been reported by many researchers as well as mood and emotional impact, which supported the statements from the course participants [7,34,51,52]. In a longer study, in which stressed and exhausted patients were rehabilitated in a natural and garden environment, the participants pointed to an important factor during the first days [36]. They felt that the whole environment had an atmosphere of safety and calmness, which brought about that their stress levels could drop and in addition that they could be in the present and start to rehabilitate.

The dual course environments, a zoo and the natural environments, were perceived as very positive. The two very different settings strengthened the wholeness of the courses and complemented each other thus giving rich opportunities for interesting and rewarding experiences. *“I experienced the course venue as 2 different environments-partly in the forest and partly in the zoo. Just being out in the woods would have been fun, but it was an added dimension to experiencing the animals undisturbed by the paying visitors. And on the contrary: if it had only been in the zoo, it wouldn’t have given as much as being out in the woods.”* This is something new that we have not seen in other studies. This may be due to the fact that zoos are rarely used in nature-based interventions. In addition, this place (Nordens Ark Zoo) is exclusive because of it being well designed and maintained and having a high ethical profile. The above can conceivably add values to three dimensions, which researchers Kaplan and Kaplan [53] argue are of fundamental importance in being able to recover capacity of directed attention, which in turn is important to be able to comprehend education: ”*being* away”, that is; leaving your everyday environment for something completely different; “*fascination*”, that is, an enchanting quality of an environment where your attention is held but not drained; and “*extent*”, that is, as being in ‘another world’, which is explorable without being overwhelming and having enough structure so you feel safe [54].

The caretakers described that they had felt ”led and guided” by the course leaders in a welcoming and safe way right from the arrival to Nordens Ark Zoo and throughout the course/courses (C1/C2). The course leaders, who were described as important parts of the environment as well, introduced the course participants into the different environments and their specific possibilities for explorations, excursions, and for activities or for “just being” to have restoration. Hence, this professional “guiding” into the environment seems to have been an important factor to open up for more rich experiences during the course. Similar effect was also reported in Sahlin and colleagues’ [7] describing nature guidings’ significant impact for individuals with exhaustion disorder to “open up” to deeper nature experiences and discoveries on nature’s fascinating details and processes. Kaplan [55] described the importance of having fascinating experiences in nature. It leads to a desire to have more of this and may attract the individual to more nature visits, which in turn leads to recovery.

During the C2, it was described as easy to introduce users to new activities that later could be included in the home activities. The Nordens Ark Zoo’s environment aroused users’ inspiration and motivation to try new activities, and to make some extra effort during the course. The caretakers believed that this would have been much more difficult to motivate the users to do in the ordinary everyday environment. *”An advantage to being in a different environment with strangers is that it is easier to do things that are otherwise difficult to do at home. The users discovered at the course that, ‘If I am going to get any sleep tonight, I need to keep fighting to reach the out-of-the-way cabin and be a part of the group.”* It had thus been possible to change some users’ negative attitudes (or lack of interest) towards nature-based activities and broaden users’ possibilities to have richer nature experiences. Pálsdóttir et al. [56] and Sahlin et al. [51] showed that participants in nature-based rehabilitation increased the amount of nature-based activities after the rehabilitation, and this persisted for several months after the treatment had been completed. However, one caretaker expressed that this change, which occurred during C2, did not bring about a lasting change towards a more positive attitude on nature excursions at home: *“The user is difficult to engage at home, but during the course he was enthusiastic and receptive. He tried new things and liked them.”* Nevertheless, at home, this user vividly and in positive wordings told people about the stay at Nordens Ark Zoo and all the activities and experiences made there. This caretaker summarized; “*Hmm, I think this was the highlight of his year. I think that it was a highpoint for him, definitely.”* He repeatedly asked if they could go back to Nordens Ark Zoo.

#### 3.1.2. Encounters with the Animals

##### Encounters with the Farm Animals

Visits to the farm animals at the zoo gave opportunity to cuddle and pet, for instance the rabbit and the sheep, and to feed the chickens and horses, thus having very close physical contacts. *“It was an enormous benefit to be so near the animals. And to visit the animals in that way, together with an educator, and to go into the enclosures with them. It was in itself quite amazing.”*

Being together with animals has been seen to promote the health and wellbeing of both old and young persons. This includes the entire chain: From health promotion to treatment and rehabilitation. The interaction between humans and animals has been shown to promote social fellowship with other animal owners as well as to being outdoors more often and to physical activity, which leads to better fitness, higher vitamin D content in the body, etc. It also appears to influence people’s environmental thinking and actions. The interaction between humans and animals encompasses a wide variety of physical, social, psychological, environmental, existential, and spiritual aspects, all of which have a positive impact on people’s health and wellbeing and lead the individual to a functional whole [9,11,15]. This has led to increasingly extensive behavioral and neurophysiological studies on relations between humans and animals. Together, they support the idea that cute, round, and infantile animal faces are potent biologically relevant stimuli, that quickly and unconsciously capture attention and elicit positive/loving behavior, including willingness to take care of the animal. These animals make us pay attention, get happy, and evoke behaviors to pet and hug the animal. Particular attention has been paid to the neuroendocrine regulation of social bonding between humans and animals through oxytocin secretion [57].

Oxytocin is a neuropeptide that, when released, has been shown to produce calming effects and to strengthen social bonds, in both animals and humans [58]. It is well known that it is released when mothers breastfeed their young children, but it is also released when humans have enjoyable communications with animals, especially when they pet and cuddle smaller companion animals, such as dogs [59]. Oxytocin is triggered primarily through tactile interaction, but it can also occur through eye contact, even in occasional encounters with animals [60].

**Figure 3:**
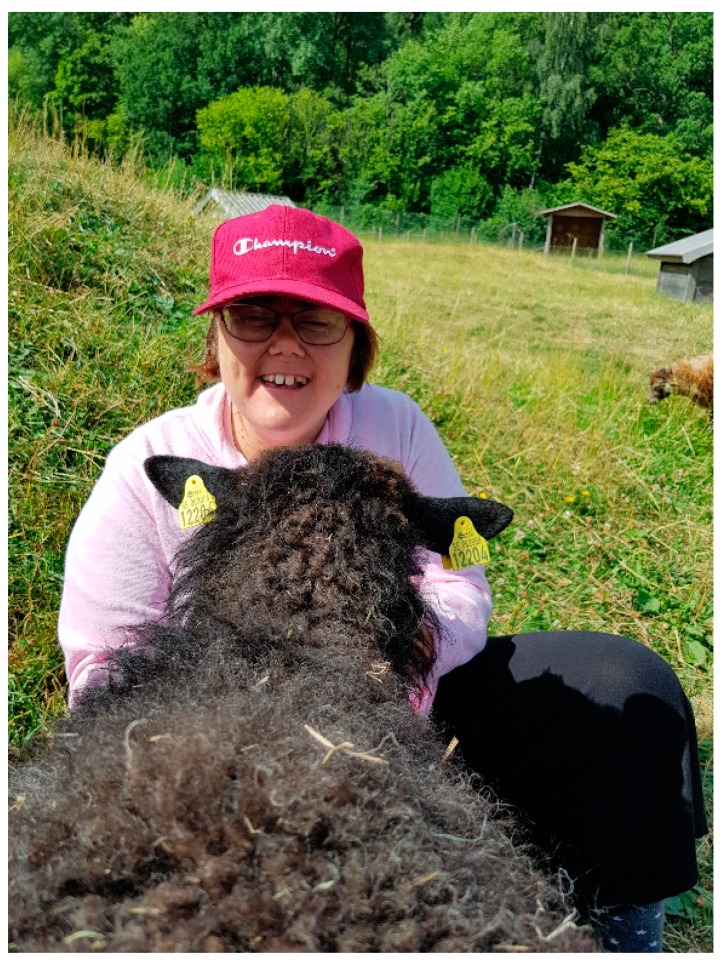
Girl feeding a sheep.

During the interviews, many memories from different users’ happy experiences in meetings with the farm animals during course days (C2) were expressed. *“We have a girl* (a user who participated in the step 2 course) *who has never been around sheep. When she sat down in the sheep pasture to feed them, and one could see how she bubbled with joy, it was quite…I don’t even know how to describe it. To see such happiness, such joy.”* (Figure 3).

The fact that farm animals can have positive effects on especially unfocused and loud children who have difficulties sitting still and have problems with concentration ability is also described in other studies [21,22]. In a review on the effect of animal-assisted interventions on children with ADHD, it is concluded that animals have positive effects on several core symptoms [61].

##### Encounters with the Wild Animals

The large enclosures, where the wild animals could easily withdraw to be distant and not be seen by the public, were appreciated. It was mentioned as reflecting the essence of Nordens Ark Zoo and its respect for the animals. Here, the animals were not primarily kept as entertainment to the visitors as expressed by one caretaker: “*It’s on the animals’ terms.”* If the animals were not seen during a walk in the park, it was mentioned as a confirmation of the respect for the welfare of the animals. During C1, a guided evening walk in the wild park during closing time was included. This was experienced as a privilege that gave the caretakers feelings of luxury and being specially chosen. The caretakers felt valued and prioritized to be involved in the close encounters with the animals and to be part of something beyond what the regular paying visitor experienced. Encounters with the wild animals in the park instilled strong emotions and fascination. *“We were allowed in after closing.// We felt really important.// It was a privilege to be a part of it.// Our own fast track into the work. And to feel that you are specially chosen and get to see this when it’s closed.”* Having the opportunity to meet a tiger in a dark autumn night’s guided walk, face to face with only a few inches of a high, safe, glass wall in between, was an overwhelming experience. One caretaker described how meeting with the wild animals gave rise to existential experiences and that this occasion also contributed to an increased understanding of how a user, who is not used to be in nature, may experience the encounter with nature. *“It is indeed very special for me to meet a tiger at 2 m distance, and it is a powerful experience that might correspond to being in the mountains up north, as well as in this unspoiled nature. I was very touched. That is perhaps the kind of stimulus* (to meet a tiger or a wolverine) *I need to understand how it can be for someone who has never been in the forest. It may give rise to the same feeling to discover the moose droppings lying on the path where we are walking. Maybe it could be something similar, maybe that could be just as great for someone else. And I need more stimulation; I need to meet a wolverine to experience this.”*

#### 3.1.3. Discovering New Possibilities in the Home Environment

The different environments provided impetus for activities in their everyday life to work together with the users. Activities and knowledge gained in the course was explained as easy to transform to any specific or different nature type back home. A caretaker from the northern part of Sweden where nature looks very different from nature at Nordens Ark Zoo explained: *“This* (the nature at home and at Nordens Ark Zoo) *are nothing alike, but, in some ways, they are the same. I gained a great insight, and I could take it back and convert it to the environment I am in. It has been very useful. So, I don’t believe it has been a problem*”.

Participating in the course and taking part in the different activities presented by the course leaders opened up to see the possibilities available in the everyday nature environments in the nearby nature at home. All informants described that they after the courses had more “open eyes” to what could be done back home. “*We use the local environment considerably more. You don’t have to go far. There is so much in the local area that you can enjoy, but you usually do not.”* The course had conveyed a conviction that nature activities do not have to be complicated or call for demanding efforts but should be accomplished in a joyful, playful, and unexacting manner *“It* (what you see in nature nearby home) *becomes more valuable. You look at your own surroundings and see more: Look there! What you see in daily life as trivial is also fantastic! That rock- of course it lays there- but it is also a really nice rock. You walk a bit more with open eyes, and what you see does not have to be so extremely remarkable.”*

### 3.2. Improved Quality in Nature Based Visits

All caretakers declared that the course to different degrees had contributed to increased knowledge relevant and useful in their work with users. Many informants stated that before the courses, they already had knowledge about nature *“I had prior knowledge, so the course gave nothing new. Still, it gave peace and quiet—undemanding participation.”* However, all added that the course nevertheless had given new inputs, ideas, tips, security, and understanding as expressed by the teacher for the younger children:

*“I was a scout when I was young, and I feel very comfortable lighting a fire and cooking. But then there were many small things I learned. I think about all the parts (in the course), and when we were looking at different flowers in the meadow and so on. I can see that: Oops! I thought I knew so much! I really thought so, but it turned out that, after all, I have a lot more to learn.”* The teacher for the younger children had limited time for developing activities to serve 65 youth for regularly nature-based activities and she commented: *“Staying in nature has always been a regular part for our children. But the course gave new ideas, which made the planned nature activities more interesting and caught the children’s attention in a beneficial way.”* Also appreciated were the possibilities to test equipment during the course e.g., binoculars, magnifying glasses, camp stove, hammock for forest/outdoor use, tarpaulin: *“we have got a better toolbox.”*

#### 3.2.1. Frequency of Nature Visits

The 12-month follow up showed that 13 (41%) of the 30 caretakers offered more frequent nature/animal-based activities to their users after the course compared to before participating in the courses. “*We have been out in the forest much more, to touch and feel different objects there, and we include much more of what the course has offered*.” and “*We stop and use our senses when we are out in nature. Day trips to the location where we can experience nature together.*”

**Figure 4:**
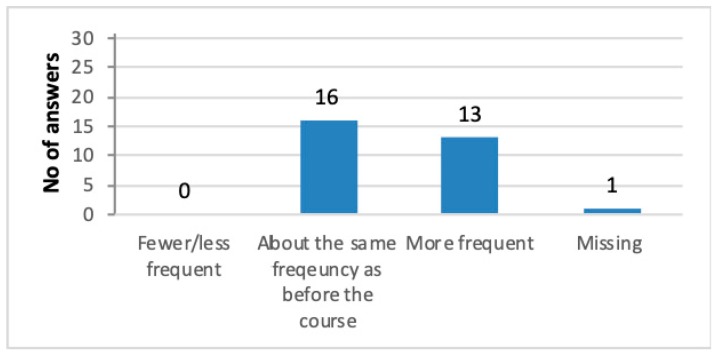
Distribution of the caretakers’ responses regarding the frequency of nature and animal-based activities after the courses. *N* = 30.

Sixteen caretakers (55%) reported about the same frequency of nature stay as before the course (Figure 4): *“It depends greatly on that it isn’t me who decides what we will do, but rather that it is largely the supervisor who is planning my user’s and my activities.”*

Another caretaker stated: *“Not activities more often, but with better quality”*, and another comment: *“It appears to me that we are more and more observant to what there is to experience in nature. We make stops more often. Then we convey the information we learned to our colleagues who did not attend the course.”*

In comments in the follow up questionnaire, as well as in the interviews, caretakers explained that nature days were in most cases included in weekly schedules that also includes other types of regular activities such as work training, working in the daily programs’ cafés, and different indoor chores. Consequently, the time available for nature activities is fixed and limited.

#### 3.2.2. Increased Content

The caretakers reported being inspired by the course to try and practice what they learned in the everyday work, which led to improved content and quality of nature visits. The safety, security, and enthusiasm gained in the courses led to the development and testing of new nature activities together with the users. Consequently, the contents of the nature-based activities were broadened and deepened, as reported by 73% of the caretakers.

**Figure 5:**
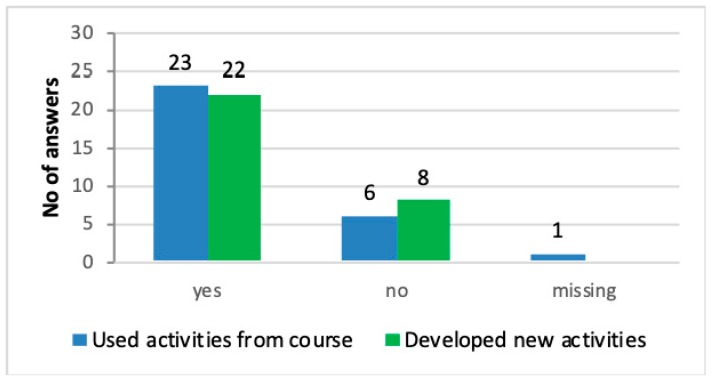
Distribution of caretakers’ reports to the questions if activities learned during the courses had been practiced with users after the course and if there had been a development of new activities. *N* = 30.

All caretakers agreed that the courses had led to quite significant changes concerning the contents of the nature visits. A majority of the caretakers (67%) stated that they after the course more often choose activities in nature and/or animals for their users. The contents during nature activity days were richer, more knowledge-based, and they included a wider variety of activities. More than three-quarters (79%) of the caretakers reported that they have continued to include activities learned in the course, and 73% reported that they had developed and designed new activities inspired by the course/courses (Figure 5). One caretaker made the following comment: *“We started a study circle during summer one day a week including the theme ‘Close to Nature’ with outdoor cooking, mixed with animal/forest themes and outdoor games and activities.”* Although the majority of caretakers had developed and broadened their nature repertoire, not all caretakers have had the same possibility due to different reasons. This is examples expressed by three caretakers: *“No, because interest is not so great, so it is difficult to motivate going out into the woods”,* and *“No, we do the same things, but with deeper thought behind the activity.”*, and *“No. I think it depends on the fact that I already knew much of what we learned in the course. Starting a fire, sleeping in a lean-to, and so on.”*

The courses did successfully convey knowledge about the positive effects of nature exposure based on research presented at the very beginning of the C1s. The scientific presentation about the effect that nature and animals have on physical and psychical health served as a base for further activities and discussions and gave a special kind of authority and lent weight to the activities following later in the course. *“To understand that there was research made* (on nature’s impact on health and wellbeing) *gave feelings of safety as well. It was reinforcement that this is actually real and right. And I believe that we have also reflected that to our users. That is good thinking. Being in nature is not fuzzy. It is good for us.”*

Some caretakers had already, before participating in the courses, included sensorial aspects of nature stays, but to a lesser extent. These had been developed and widened because of the course. The caretakers opened up to include and to stress sensorial experiences, that is, to use all senses to explore during nature walks in their work. Similar descriptions were reported in Sahlin et al. [7]. Participants in an NBI were positively affected during nature walks where a nature guide pointing out details and processes in nature and opened up the participants’ minds to fascinating sensorial impression. The beneficial impact of birdsongs in nature for stress reduction and wellbeing has been reported by Ratcliffe et al. [62] and Hedblom et al. [63]. Activities from the course such as perceiving the forest’s fragrances, listening to the wind rustle in the treetops and birdsongs, and observing details and differences in, for instance, different trunk textures excited users’ curiosity and encouraged to seek further knowledge using books and internet. This was especially outspoken in interviews with one team of caretakers, and also by the teacher for the younger children.

Activities including animals were seldom mentioned in the 12-month follow up questionnaires; one exemption was: *“We usually have summer activities; for example, traveling to an urban farm with livestock.”* Animal activities were described in the interviews as more difficult to carry out, especially activities including wild animals. Some caretakers working in group-based daily programs brought their own dogs, which became popular participants during the regularly nature walks. One daily program was involved in organized dog walks and a dog day-care. Three of the caretakers offered services at their private farms where the interventions included support by farm animals, mostly horses and equestrian therapy. Equine therapy has been shown to improve character skills such as thinking flexibly, taking responsible risks, managing impulsivity, persistence, and listening with understanding and empathy in youths during a three-month intervention [64]. It also seems effective in improving social function in people with ASD [13]. However, tips from the course leaders on how to “fake” wild animal encounters during nature walks were mentioned in the interviews as being successfully practiced. It could be to “prepare” the planned nature visits by purpose by placing objects (e.g., a varnished moose dropping, antler, a dried snakeskin, or a mouse skull) in advance on the path in order to surprise, inspire to discussions, explore, and tickle curiosity, thus making the nature walk more attractive.

### 3.3. Bringing the Knowledge Back Home

A secondary effect was that participating in the courses led to the spreading of knowledge on nature’s impact on individuals’ health and wellbeing to colleagues, supervisors, politicians, and other professionals working. *“And I was the only one from my team who participated in this course, so I had to share my knowledge (from C1) to my colleagues. So, it’s not like, when I’m working, she’s outside, otherwise she’s indoors. It has become more automatic that all caretakers go out more. They have also had their eyes opened to, ‘yes, this can actually be done!’ So, this is, of course, very good. She has many more outdoor activities.”* In this way, nature visits also became more frequent to other users in that particular program. One team of caretakers inspired colleagues to start two new nature groups, giving more users the possibilities to have nature experiences. This team also informed supervisors and politicians in the Social Welfare Department in their town, which was met with interest and curiosity. Another team of caretakers had arranged a teachers’ seminar-day for their co-workers to inspire and inform about including nature in education. One caretaker had participated in a conference lecturing about her experiences based on the C1 *“so we stood there (me and my boss) and represented this course with the intention of opening their eyes. To inspire more caretakers and other health professionals to attend the course as well.”* A third team of caretakers working with individuals on very early development levels described that every user had their own individual personal caretaker (no participants in C1/C2) accompanying on nature activity days. These caretakers showed increased interest in understanding nature more and developing new ideas to use in their work. Two of the caretakers’ teams have after the course arranged camps at Nordens Ark Zoo every summer for users and some had arranged with Nordens Ark Zoo for one-day excursions and visits to Nordens Ark Zoo with users.

### 3.4. Wellbeing and Health

Nature environments are often described in the literature to be restorative [7,34,51,55,65] and thus give individuals several beneficial physical and mental health effects. White and colleagues [66] recently reported that 2 h of recreational nature visits a week had a significant beneficial self-reported effect on wellbeing and health. The peak effect was reached with 200–300 min in nature. The time spent in nature for most of the users and caretakers in this study was reported to be 2–3 h (morning hour to lunchtime) of exposure to nature on a weekly basis and should therefore be of sufficient length to obtain the good effects reported in the research. The effect of daylight when in nature is also reported to be a likely contributing cause of the beneficial effects reported on wellbeing, mood, and stress-related health [67].

Longo et al. [68] have identified a series of common constructs of wellbeing including happiness, vitality (energy), calmness, optimism, involvement (engagement), self-awareness, self-acceptance, self-worth, competence, development, purpose, significance, and self-congruence (harmony, balance). Positive feelings, positive functioning, and pleasure could be added to the list. The majority of these constructs are described in the caretakers’ narratives as effects from the course. They are more or less related to the influence of being in nature during the course as well as in increased activity in nature back home in the everyday life. Therefore, it is reasonable to conclude that wellbeing as well as health aspects are positively influenced by the course through the increases in the caretakers’ nature activity repertoire.

#### 3.4.1. The Users’ Wellbeing

**Figure 6:**
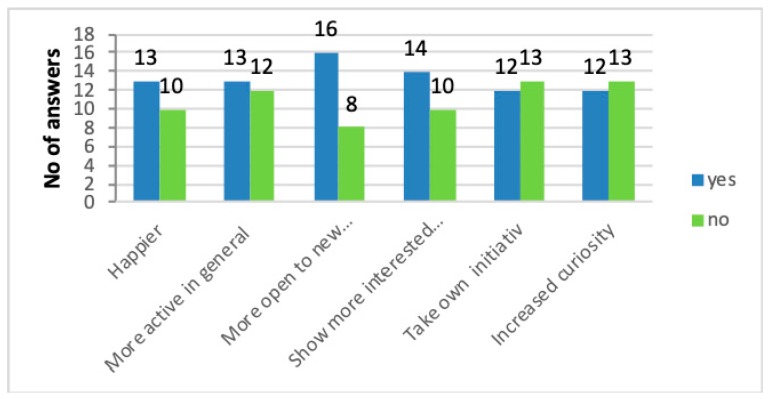
Caretakers’ ratings of wellbeing aspects after users’ participation in nature activities. *N* = 23/25.

Between 46% and 67% of the caretakers reported users’ improved mood, showing more inspiration and activity levels in the 12-month questionnaire. The users were experienced as happier (54%), more curious and active (50%), more interested in general (50%), and more open to new activities (67%) after participating in nature and/or animal activities. Forty-eight percent of the caretakers stated that their users after having participated in nature-based activities more often presented own suggestions and ideas about favorable activities to do (Figure 6). The users were described as having developed more curiosity for details and had become more observant during nature-based activities, which may be partly explained by caretakers’ greater consciousness: *“After attending the course, I have discovered that, when we are out in nature, the users are more curious in small details. This can probably be explained by our own increased knowledge and our being more perceptive. We stop more often and really look around us// The benefits that come and have another type of fitness, not just the physical.”*

One caretaker described how her user, because of a severe neurological disease, was constantly troubled by involuntary (bodily) movements, which disturbed sleep or even made it impossible to sleep. However, after nature walks, the user showed better mood, was more relaxed, movements were significantly less, or even none at all, which gave the user relaxation and restoration, and a nature walk was often followed at home by a healthy and good sleep. *“She has become more relaxed, much alert and more engaged when we are outside. And when she comes in, she can sleep for a while and rest.”*

Being able to cope successfully with new challenges during the C2 evoked users’ pride and improved self-confidence and self-esteem, which increased feelings of wellbeing for the users but also for the caretakers who observed and thus rejoiced for the users’ success and development. Being in nature regularly in the users’ everyday life was expressed by the caretakers as very important for the users’ wellbeing. Nature conveyed a sense of undemanding environment and acceptance for being just the way you are. It was described as making it easier to have conversations, between user and caretaker, about deeper issues during walks in nature compared to during activities indoors. This also added to users’ increased senses of connection, self-congruence, and involvement, and thus improved wellbeing. *“For our users, above all else, it is very important to have this release valve. To come and just be outside, without so many demands. More that: we are here now, and we will be comfortable. – (being in nature) also inspires many important conversations of emotional character.”*

Linked to users’ increased wellbeing were several issues expressed by the caretakers such as increased curiosity, improved mood, stress-relief, and also positive social interactions and relations due to nature activities. Nature was described as undemanding and permissive, and the visits and activities performed there gave rise to fewer failures hence strengthens the users’ self-confidence as expressed in one narrative: *“We undertake considerable job training, and there is a quite different focus* (than in nature) *where you must perform the tasks precisely and properly, empathize with customers and everything. So it is, it is neutral ground to be out* (in nature). *// this is probably so significant for people in order to be able to cope. Having the energy to move on according to Maslow’s hierarchy of needs, realizing oneself one must feel good. And how to feel good? Well, you can, if you are given enough room to breathe//and you don’t fail in that room* (nature) *rather you gain self-esteem. There are myriad failures* (in daily life). *But this, that we have nature and simplicity, that is the best.”* There are clear connections that show that the longer you stay in natural environments per week, the lower the stress levels [69,70]. Spending at least 120 min a week in nature is associated with good health and wellbeing [66].

Hence, many positive mental and physical health effects derived from nature activities were reported, which are in line with a rich body of published results [1,2,3,4,5], all of which report clear links between increased exposure to natural environments and improved mental and physical health and wellbeing; better sleep quality; improved mood and quality of life; and increased motivation and function regarding dealing with everyday activities.

#### 3.4.2. The Caretakers’ Wellbeing

The environment at Nordens Ark Zoo conveyed feelings to the caretakers of “being in a bubble” of tranquility, which enabled them to leave stress and demands from everyday life, hence making it easier to open up to what the course offered: *“I experienced a sense of calm as soon as we got there, it’s the only way I can summarize it. My body knew what and how it could feel there.”* Thus, mediating positive feelings of being embraced and held by nature, and of obtaining mental and physical rest and recovery, which contributed to the increased wellbeing described by the caretakers [36,71].

The environmental effects of tranquility during the courses were also described as having lasting effects, and feelings of flashbacks could be evoked at home by simple thinking or talking about the course days at Nordens Ark Zoo, thus giving moments of restoration and stress relief during daily chores. Participants in nature-based interventions have reported similar supportive flashbacks of stress-reducing nature experiences [51].

Caretakers’ participation in the course led to greater security of being in nature and thus increased self-confidence in one’s own ability. However, there was a slight difference between the two groups of caretakers. Those working in teams in the public sector declared that they already before the course mostly felt secure and safe when being in nature but gained more of this during the course. Caretakers working alone with a user, for instance as personal caretakers, had acquired a more solid sense of security and safety for being out in the forest during the course. *“Yes, now I feel more confident. I venture more. Now I know how to start a fire, or how I will find my way, which way to go, because we had a lot of orienteering in the course.”* This inspired them to expand the nature activities both for their user/users but also for their own private nature experiences. The personal caretakers seemed to have more freedom in their weekly schedule to expand the frequency of nature activities.

The improved wellbeing linked to nature-based activities among the younger children/pupils was based in social development, self-development, and testing of one’s strength in play and activities in the forest. The teacher’s narrative included descriptions of the smaller children making new friends more easily in the forest compared to on the schoolyard. More silent, shy, and withdrawn children could blossom and thus receive respect from the other children for knowledge that had earlier been “hidden.” The ability to work together towards a goal improved and a more playful attitude towards testing and accepting failures was seen. Although this teacher already during long time had worked with nature-based activities for the very young children, she declared that the course had given new perspectives and a larger activity repertoire.

Participating in the nature and animal activities during the course gave feelings of increased wellbeing. Several interviews describe how the course had led to more joy and feelings of contentment when being in nature and statements of nature’s mediating several positive mental health effects for the caretakers as well as for the users such as inspiration, increased wellbeing, and less stress. An important effect highlighted by all caretakers was that the courses led to sustainability in the caretakers’ work life.

#### 3.4.3. Sustainability for the Individual

Essential to the course participation was to meet with others in the same profession to exchange experiences. This was expressed among both caretakers with more nature experience and those with less experience. All caretakers described that they gained confirmation that the nature-based activities they offered their users was in line with what other course participants did. *“Also, the courses inspire us to still be committed and really believe in what we are doing.”* It contributed to reassurance and confirmation that they performed their work well and with a good content that provided feelings of satisfaction, self-worth and self-competence, and being “good enough.” This is an important conviction for having sustainability in work life. Nature mediated several positive mental health effects for the caretakers as well as for the users such as pleasure, increased wellbeing, and less stress, which are all important ingredients to a sustainable working life and private life.

**Figure 7:**
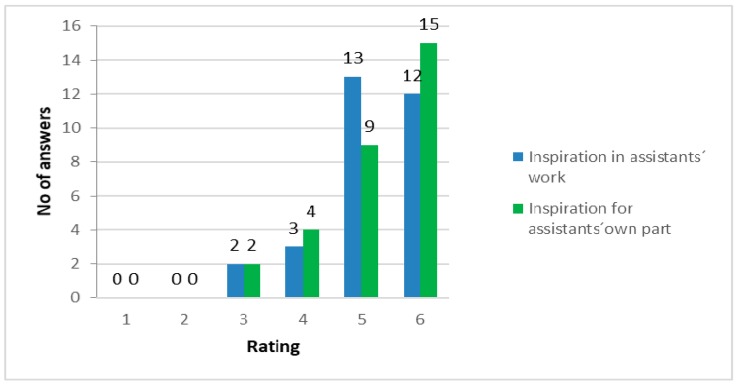
Distribution of caretakers’ ratings to the course importance for inspiration and joy in their work and for their own part. *N* = 30.

A large majority (83%) of the caretakers rated the highest values 5 or 6 regarding the impact of the course for inspiration and joy in their work giving the median value 5 (Figure 7). Half of the caretakers (53%) reported that they were more outdoors in nature in their private life after the courses. *“It was a confirmation, a good input so that we get the energy to endure a little while longer and continue to give.”* and *“But what has become very obvious is that primarily we have our private lives, where we choose a lot of nature ourselves to cope with work and so. For me that goes without saying.”* In the interviews, the caretakers described the benefits from the inspiration and joy that the courses contributed in their working life. *“For me, personally, the largest effect of the course has been the message that it (taking users outdoors in nature) doesn’t need to be so extraordinary. I venture more // before, I felt, “But I don’t have the knowledge to work with forest and field and wild animals.” I didn’t dare to involve them in the way I do now.”* All caretakers highlighted that participation in the course had led to a more sustainable working life. Several caretakers described that the courses had given them inspiration and the much-needed energy to continue in the profession. *“I feel that you got an amazingly good input again, because you need that. You constantly give, but rarely get anything back for yourself. So, I feel that this allowed you to refill with everything, really. Maybe not just skills, but that you also got refilled energy.”*

Sahlin and colleagues [51] and Pálsdóttir and colleagues [56] found similar results on increased frequency of nature activities following participation in nature-based interventions. Also, sustainability for the users’ situations and every day work was mentioned where nature could mean restoration and loading of batteries to be better equipped to handle everyday demands. *“And now I feel that when we have a week with our users, there ought to be a rule that they get a day every week in nature to restore, in order to handle all the tasks that they otherwise have in their weekly schedule.”* (different work in the users’ weekly activity schedule other than nature days).

#### 3.4.4. Relations

There were several statements in the narratives that because of participating in C1 (participation without user/users) relations between caretakers and their users had improved and deepened. One personal caretaker working with a severely neurologically disabled woman with the following statement described this: *“My job has taken on a whole different meaning in that we are outside so much more. You get so much out of it. I think it is so incredible, that she cannot talk, but we can still have so much in common by just walking out the door. It is so amazing that such a little thing, like going out and looking at the trees or looking for footprints in the snow, can bring us so damn tight together. I, as a caretaker, and she, as my user. That has enabled me to read her in a completely different way than before the course.”*

In addition, relations and group atmosphere indoors were affected because of nature visits. All caretakers’ narratives stated that there was a calmness in the users’ groups during days that include nature activities, but not during the indoor days. There were fewer conflicts between the users in the nature groups during outdoor activities compared to when they had indoor activities. This atmosphere following nature activities also affected the rest of that day and promoted good social behavior and calmness in the subsequent indoor activities, thus contributing to increased experienced wellbeing but also having a positive effect on social relations with the other users who were not participating in the nature program.

Some of the caretakers stated that they have had preconceived opinions of what could be accomplished with regard to nature-based activities due to users’ disabilities and difficulties. However, new ideas and possibilities were discovered and tested. The caretakers described that the experiences in the environments at Nordens Ark Zoo made them more disposed and willing to try and to adopt new activities to use in their nature activity repertoire at home. *“At home, we* (the caretakers’ group) *would have definitely said, ‘No, it is not possible! Out there* (at Nordens Ark Zoo), *when it* (what were tested and experienced) *was done, we felt, ‘I did it! I could!’”* The caretakers stated that actually practicing the nature-based activities during the C1, learning by doing, was important to gain the security and feelings of safety to include new activities at home. The more developed nature-based activities at home was also mentioned as contributing to deeper relations between caretakers and their users. *“We feel more able and inspired ourselves, which is passed on to our students. To do something together in nature has been successful in many ways, in building the relationship between us, and between students* etc. *You gain a stronger self-esteem when you succeed at something.”*

The courses had opened up to a broader insight on what is possible to do and to be bolder to “trial and error” instead of deciding in advance that an activity or a program is not possible to perform because of a person’s disability. *“I have really felt after the course that sometimes one thinks, ‘but it is not possible because she is in a motorized chair.’ Or, ‘this is impossible to do’ … unconsciously, I had an excuse because you usually don’t do those things* (with a disabled person). “*But she can be just as actively involved as any another despite that she is in an electric wheelchair*.”

The narratives revealed caretakers’ discoveries on users’ unexpected broader abilities and caretakers own failures to recognize this before the C2 in the ordinary every day work and how a new view on users may develop: *“…there is a value to have done something like sleeping in a lean-to // you challenge yourself, but you also see a strength, you see the health benefits in the users which really makes us equal as well. Everyone has fears, so I really admire that in this gang that still has a disability, and they are so cool.”* Such discoveries promoted the caretakers to be bolder in challenging their users in activities at home. There were also reflections on the significance of having memories together, and the accomplished challenges experienced together during the C2, which seemed to enhance the relations.

## 4. Conclusions

All the results of this explorative study show that the nature and animal courses at Nordens Ark Zoo, including a two-step intervention involving caretakers and users, may reinforce the use of nature in caretakers’ regular work as well as in their private life. However, the use of animals was limited. The positive effects in the everyday work had conduced caretakers to use nature activities more intentionally for their own stress reduction, enhanced wellbeing, and recharging one’s “batteries.” The frequency of nature stays was often not possible to increase but the described improved quality included a more conscious exploring and sensory experiencing during nature stays and were one obvious result of the courses. The interviews as well as the 12-month follow up showed in unison that the content in the planned nature activities after the courses enhanced the users’ wellbeing, mood, self-confidence, interests, curiosity, and activity levels, thus confirming the theories on nature exposure as supportive for these dimensions.

One conclusion that can be drawn from the caretakers’ narratives was that they experienced themselves as a disadvantaged professional group working with users with special needs (disabilities) with regard to lack of further education but who felt that they are in great need of that for a sustainable working life. Therefore, the courses gave new energy and inspiration to continue to give out to others and stay in the profession.

The results from this study give strong indication zoos like that Nordens Ark Zoo can be used successfully for education as described in this study. We have shown the potential in combining the zoo environment with nature experiences, giving a good opportunity for nature-based interventions at zoos.

The authors’ recommendation is that for this group of professionals, more nature- and animal-based education suitable for their users’ groups should be more frequent (offered) to provide opportunities for a disadvantaged group to have the positive effects of which most other groups have obvious access to.

## Figures and Tables

**Table 1:** The number of caretakers participating in the 12-month follow up, interviews, or/and the C1^1^ and C2^2^ and the type of activity.

Type of Activity	Number Participants 12—Month Follow Up	Number of Group/Single Interviews	Number of Informants in Interviews	Participated Only in C1^1^	Participated in C1 and C2^2^
Municipal daily programs for individuals with special needs	13	5	9	2	11
School/pre school	3/1	1/1	2/1	1/1	2
Special school for mentally handicapped children	2			1	1
Personal assistance to individual with special needs	8	2	2	4	4
Farm/ equine therapy	3	1	1	3	
Total	30	9	15	12	18

C1^1^ = the first step, the 3-day course. C2^2^ = the second step, courses for caretakers and users together for either a 1-day or a 2-day course.

**Table 2:** The four main themes and nine subordinate themes from the interviews.

Main Themes	Subthemes
The impact of the environment	The course environment Encounters with the animals Discovering new possibilities in the home environment
Improved quality in nature-based activities	Frequency of nature visits Increased content
Bringing the knowledge back home	
Wellbeing and health	Users’ wellbeing Caretakers’ wellbeing Sustainability for the individual Relations
